# A Snapshot of Gay-Straight Alliance Clubs and Student Well-Being in Western Canadian High Schools

**DOI:** 10.1177/08295735231170337

**Published:** 2023-05-02

**Authors:** Maria Di Stasio, Lauren Alston, Jason Harley

**Affiliations:** 1 MacEwan University, Edmonton, AB, Canada; 2 University of Alberta, Edmonton, Canada; 3 McGill University, Montreal, QC, Canada; 4 Research Institute of the McGill University Health Centre, Montreal, QC, Canada

**Keywords:** gay-straight alliance, high school, self-determination, bullying, depression

## Abstract

Gay-Straight Alliance (GSA) clubs promote safer school environments for students. GSAs typically refer to student-led, teacher-supported school clubs that serve youth of diverse gender identities and sexual orientations. This study investigated the relationship between students’ awareness of school-based GSAs and their bullying experiences, mental health, self-determination, and relationships at school and home. Findings showed that LGBTQ2S+ students experienced higher rates of bullying and symptoms of depression and scored lower on self-determination subscales than cisgender heterosexual students. Interestingly, students who were aware of their school’s GSA club scored higher on the self-determination subscales regarding family relationships and lower on bullying compared to students who were unaware of their school’s GSA club. LGBTQ2S+ students had lower rates of comfort with their sexual orientation at home and school than their cisgender heterosexual students. Implications and future directions are discussed.

## Introduction

Throughout Canada, youth of diverse sexual orientations and gender identities have advocated for safer spaces in their schools for decades (McMillan, 2019). Many school environments are still unsafe or not inclusive of students who are lesbian, gay, bisexual, trans, queer, questioning, Two-Spirit, asexual, intersex, non-binary, and other identities (further referred to as LGBTQ2S+ youth). A recent EGALE Climate Survey on Canadian schools reported that 48% of LGBTQ2S+ students felt unsafe at school, compared to only 4% of cisgender heterosexual students (Peter et al., 2021). Overall, LGBTQ2S+ students experienced more significant verbal harassment than cisgender heterosexual students regarding their sexual orientation, gender identity, and gender expression (Cénat et al., 2015; Peter et al., 2021).

Gay-Straight Alliance (GSA) clubs, also called Gender and Sexuality Alliance, are common LGBTQ2S+ school-based supports. Over the past decade, there has been an increase in GSAs across North America (Peter et al., 2021). Qualitative research involving LGBTQ2S+ student perspectives has demonstrated that GSAs positively influence the physical, social, emotional, and academic well-being of LGBTQ2S+ youth and allies (Porta et al., 2017). GSAs provide an opportunity for youth to be members of a community, to connect, support emotionally, and belong, along with opportunities for leadership and fulfillment of needs (Porta et al., 2017). A comparative analysis by Fetner and Elafros (2015) revealed that LGBTQ2S+ students had more support from teachers and administrators in schools with GSAs than in schools without GSAs.

During adolescence, many students struggle with their identity and the stress of social pressures. This can be especially true for LGBTQ2S+ youth (Russell & Fish, 2016). Socio-contextual factors can facilitate the expression of identity for LGBTQ2S+ youth with autonomy-supportive contexts (Legate et al., 2012) and increase students’ self-determination. To our knowledge, no research has examined the impact of GSAs on students’ self-determined behavior and levels of comfortability with their gender identity and sexual orientation. This research investigated how students’ awareness of their school’s GSA is associated with self-determination (i.e., feelings of autonomy, relatedness, and competence), their relationships at school and home, safety, and symptoms of depression.

### Gay-Straight Alliances (GSAs) in Schools

In response to homophobia and transphobia in schools, LGBTQ2S+ students and their allies have created student groups (e.g., GSAs) to combat victimization, promote well-being, and create safer spaces on campus. GSAs have been argued to be one of the few opportunities for students to contribute to political change within their education system, impacting all students regardless of their gender identity or sexual orientation (Herriot, 2015). The broad influence that GSA clubs have on the school environment is why our research includes the entire student body.

Researchers have focused on GSA clubs as moderating factors contributing to reducing LGBTQ2S+ victimization in schools. A systematic review indicated that the presence of GSAs in schools was associated with lower levels of school-based victimization of LGBTQ2S+ youth and lower levels of fear for safety for all students (Marx & Kettrey, 2016). Even schools with harmful environments characterized by reported feelings of unsafety, lack of school connectedness, and low support from school staff had lower rates of reported homophobic bullying among LGBTQ+ and heterosexual, cisgender students when a high-functioning (based on student subjective ratings of club effectiveness) GSA was present (Ioverno & Russell, 2020). Li et al. (2019) reported school-wide benefits where they found long-lasting protective effects of GSAs on the school climate, positively impacting all students. Such findings suggest that the presence of a GSA club within a school is associated with a positive school climate, and even if students do not actively attend, knowing that the club exists may provide comfort.

In Western Canada, where our research is situated, the popularity of GSA clubs in schools increased throughout the 2010s. Researchers have made significant contributions to the field by addressing mental health, victimization, empowerment, and other social factors in school-based GSA clubs (Ioverno & Russell, 2020; Li et al., 2019; Marx & Kettrey, 2016). Our study aimed to build on previous literature by investigating how students’ knowledge of the presence of a GSA club in school influences their self-determination. By adding self-determination to other measures (i.e., bullying, comfort with self, and symptomology of depression), we contribute a unique perspective to GSA research. Based on previous literature, we gather that GSA clubs are designed to help LGBTQ2S+ students feel accepted while providing a space for self-expression and the development of identity and self-determination.

### Self-Determination Theory

During adolescence, exploring and committing to an identity is part of the individual’s psychosocial and personality development (Erikson, 1980). When individuals have a sense of identity, they feel like they fit in and belong. Social-contextual environments that support and facilitate this process of identity formation will increase students’ agency and self-determination (Legate et al., 2012). Individuals become self-determined when their needs for competence, relatedness, and autonomy are met (Deci & Ryan, 2000). This research explores GSAs from the lens of self-determination theory, which proposes that an individual’s psychological needs for relatedness, autonomy, and competence contribute to human motivation and are essential for growth, integrity, and wellness (Deci & Ryan, 2000). Researchers have demonstrated a positive association between satisfying psychological needs (i.e., autonomy, relatedness, and competence) and identity formations (i.e., multiple dimensions of commitment and exploration; Luyckx et al., 2009). GSAs can be supportive contexts that satisfy psychological needs and provide students with the tools to challenge homophobia and transphobia in their schools. For example, LGBTQ2S+ youth are more likely to disclose their sexual orientation or express their gender or non-binary identity in autonomy-supportive contexts. These contexts are associated with more positive well-being (Legate et al., 2012).

Engagement with a GSA can predict increased hope, self-efficacy, and perceived peer validation among high school students (Poteat et al., 2020). Even if students do not participate, their awareness of their school’s GSAs has its benefits. For example, GSAs can impact the school climate by improving inclusivity, increasing awareness, and reducing bias and discrimination against LGBTQ+ students (Greytak et al., 2016). Student awareness of their school’s GSA club provides a sense of community, emotional connection, and social support (Porta et al., 2017). Considering the positive impact of GSA clubs on students, investigating aspects of self-determination on the awareness of school-based GSAs would provide insight. In this paper, we examine students’ awareness of the GSA club in their school, and not necessarily attendance in GSAs, because having an awareness of GSAs is enough to impact students’ well-being, as is shown in the literature.

### The Present Study

Teachers have pushed for resources and social support like GSA clubs in schools across Alberta, Canada. As the demand for LGBTQ2S+ and GSA resources increased, the Alberta Ministry of Education passed several bills (Bill 10, Bill 24, and Bill 8) that changed the legal parameters of GSAs clubs regarding the resources allocated to the clubs, students’ rights to privacy and student control over who knows about their identity. With the changes in legislation highlighted in the media (Clancy, 2019), an updated understanding of students’ experiences regarding awareness of GSAs can contribute to evidence-based programming and inform policies that reflect the community’s needs.

Our study examined LGBTQ2S+ and heterosexual cisgender students’ well-being by assessing their levels of self-determination, experiences with bullying, symptomatology of depression, and comfort with expressing their sexual orientation and gender identity. To date, no research has examined high school students’ basic psychological needs (i.e., autonomy, competence, and relatedness) for self-determination and its association with GSA clubs in schools. We collected survey data and demographic information from students in public junior and senior high schools in Alberta, Canada. Within the demographic portion of our survey, students were asked about their awareness of their school’s GSA club and their feelings of comfort with their sexual orientation and gender identity at home, at school, and with their friends. Based on our objectives, the research questions were as follows:

- Research Question1: How comfortable do LGBTQ2S+ and heterosexual cisgender students feel with their gender identity and sexual orientation at home, school, and with their friends?
- Research Question2: Are students’ awareness of GSAs related to their well-being regarding experiences of bullying, symptoms of depression, and self-determination?

## Method

### Participants

Two public school boards in the province of Alberta participated in the study. Urban schools were selected by geographical region so that the sample was drawn from different areas in the central city of Alberta. Principal consent was attained, along with teacher consent who volunteered with their classroom to participate in the study. Parental consent was required for students to participate in our research. The total sample consisted of 169 student participants in junior high school, grades 7 to 9 (*n* = 17), and senior high school, grades 10 to 12 (*n* = 152) from seven schools in one city. The student participation rate was 10.6%.

### Measures

#### Descriptive Statistics

The survey included students’ demographics, ethnicity, gender identity, and sexual orientation.

#### Self-Determination

Self-determination was assessed using The Self-Determination Basic Psychological Needs Satisfaction Scale (La Guardia et al., 2000), which is comprised of 21 items measuring the psychological needs of *autonomy* (7 items), *competence* (6 items), and *relatedness* (8 items). Each item was rated on a 7-point Likert scale ranging from 1 (not at all true) to 7 (very true). The published Cronbach’s alpha for the overall Self-Determination Basic Psychological Needs Satisfaction Scale was .85 (Guo, 2018) and .89 for our sample. For the Self-Determination Basic Psychological Needs Satisfaction subscales, the published Cronbach’s alpha is .76 for autonomy, .74 for competence, and .77 for relatedness (Guo, 2018). In our sample, Cronbach’s alphas were: .69 for autonomy, .66 for competence, and .83 for relatedness.

The Basic Psychological Needs Satisfaction in Relationship Scale (La Guardia et al., 2000) was administered three times to measure basic interpersonal psychological needs in relationships with peers, teachers, and at home. The scale included 9 items (autonomy = 3 items, relatedness = 3 items, and competence = 3 items). Each item was rated on a 7-point Likert scale ranging from 1 (not at all true) to 7 (very true). The published reliability of the Basic Psychological Needs in Relationships Scale is .92 for mother, .92 for father, .92 for romantic partner, and .90 for friends (La Guardia et al., 2000). In our study sample, the reliability coefficient for each subscale of the Basic Psychological Needs in Relationships was .88 for friends, .87 for teachers, and .90 for family.

#### Bullying

The Illinois Bullying Questionnaire (Espelage & Holt, 2001) was used to assess bullying and victimization. The scale has a total of 17 items and includes three subscales: bullying (9 items), victimization (4 items), and fighting (4 items). The scores range for the bullying subscale is 0 to 36, the victimization subscale is 0 to 16, and the fighting subscale is 0 to 16. A total for each subscale was calculated. The published Cronbach’s alpha for bullying, victimization, and fighting is .87, .88, and .83, respectively (Espelage & Holt, 2001). In our sample, Cronbach’s alpha was .80 for bullying, .81 for victimization, and .77 for fighting.

#### Depression

The Center for Epidemiological Studies Depression Scale (Radloff, 1977) was used to assess non-clinical levels of depression. The scale consists of 20 items that measure symptoms of depression in the last week. The range of scores is 0 to 60, with higher scores indicating the presence of more symptomatology. The Center for Epidemiological Studies Depression Scale is reported to have a reliability of Cronbach’s alpha of .85 for non-clinical populations (Radloff, 1977). In our study population, Cronbach’s alpha was .74.

#### Comfort With Identity

Participants were asked six questions regarding how comfortable they feel expressing their gender at home, at school, and with their friends, as well as how comfortable they feel with their sexual orientation at home, at school, and with their friends. A Likert scale ranging from (1) strongly disagree to (5) strongly agree was used to record responses.

### Procedure

All measures were administered by the principal investigator and research assistants at each school site. Participating students were gathered in a classroom, and research assistants used a scripted verbal protocol to explain the purpose of the study and the terminology used in the survey. Students completed the survey questionnaire online using REDCap, a secure online Canadian data-managing survey program. Students received a link via their school email to complete the survey. The survey was approximately 20 minutes and included demographics and questionnaires on self-determination, bullying, depression, and comfort with sexual orientation. Only students with consent were able to access the survey. Students could withdraw from the study if they experienced any distress. At the end of the survey, a list of resources for counseling and social support was provided to students.

### Analyses

To answer the research questions, *T*-tests and ANOVA were used. All participants who did not identify as cisgender also did not identify as heterosexual; therefore, for our statistical analyses, we grouped non-cisgender and non-heterosexual students under LGBTQ2S+. We understand that the experiences of gender identity and sexual orientation are separate and distinct; however, we wanted to honor and include the experiences of trans and non-binary students relating to their sexual orientation in our study. Our sample of trans and non-binary students was too small to run separate gender-based analyses; however, their sexual orientations still warranted their inclusion within the LGBTQ2S+ group.

To answer the first research question, we ran a *t*-test comparing the means of how comfortable students felt at home, at school, and with their friends regarding their sexual orientation. The variance between groups (LGBTQ2S+ vs. heterosexual cisgender) was significant only for their comfortability scores at home, yielding a Levene’s Statistic of *F* (1, 165) = 8.78, *p* = .003. Therefore, in the results section, we report the *p*-value of the comfort at home scores under the assumption of unequal variances. To answer our second research question, we ran an ANOVA to determine whether there were any main effects or interactions between identity (LGBTQ2S+ vs. heterosexual cisgender) and GSA awareness (aware vs. not aware) on how participants experienced bullying, depression, and self-determination. The variance between groups was significant for victimization scores (Levene’s statistics *F* (3,132) = 9.57, *p* < .001) and the Self Determination Scale Teacher Relationships Relatedness subscale scores (Levene’s statistics *F* (3,132) = 2.85, *p* = .04) therefore we report the p-value under the assumption of unequal variance.

## Results

### Descriptive Statistics

Percentages for gender identity, sexual orientation, and ethnicity are presented in Table 1.

**Table 1.** Descriptive Statistics.

	Gender identity (%)
Trans	0.6
Trans man (specified)	1.2
Non-binary/gender fluid	1.8
Man/boy	42.8
Woman/girl	53.6
	Sexual orientation (%)
Gay	2.4
Lesbian	0.6
Bisexual	15.4
Pansexual	2.4
Queer	1.8
Asexual	1.8
Questioning	1.2
No label (not heterosexual)	0.6
Heterosexual	74.0
	Ethnicity (%)
First Nations, Métis, and/or Inuit	8.3
White, European, and/or Scandinavian	63.3
Black, African, and/or Caribbean	6.1
Latin American and Indigenous South American	5.3
Arab	3
Chinese, Filipino, Southwest Asian, and Polynesian	10.1
South Asian	10.1
Not listed	6.5

*Note*. The percentages add up to more than 100% since students could select more than one ethnicity and sexual orientation.

### GSA Awareness and Attendance

For LGBTQ2S+ students, 85.7% were aware that their school had a GSA club, and 36.1% attended the school’s GSA. Comparatively, for the heterosexual, cisgender students, 63.7% were aware of their school’s GSA club, and 3.8% attended their school’s GSA.

### Comfort With Sexual Orientation and Gender Identity

An independent samples t-test revealed differences between LGBTQ2S+ and heterosexual, cisgender students regarding their comfort levels with their sexual orientation at home and school. LGBTQ2S+ students expressed significantly lower levels of comfort with their sexual orientation at home (*p* < .001) and at school (*p* < .001) compared to heterosexual, cisgender students (see Table 2 for means). There were no significant differences between the gender-based comfort levels of LGBTQ2S+ students and heterosexual, cisgender students in the home, at school, or with friends.

**Table 2.** Means for Comfort with Sexual Orientation.

	At home (*SD*)	At school (*SD*)
LGBTQ2S+	3.71 (1.24)	3.50 (1.09)
Heterosexual and cisgender	4.66 (0.94)	4.56 (0.99)
*p*-Value	<.001	<.001

*Note*. Student mean scores of comfort level with their sexual orientation at home and at school.

The percentage of LGBTQ2S+ students who responded that they agreed or strongly agreed to feeling comfortable expressing their gender identity was 81% at home, 82.9% at school, and 97.6% with friends. We also looked at the percentage of LGBTQ2S+ students who responded that they agreed or strongly agreed to feeling comfortable with their sexual orientation at home (69.0%), at school (89.5%), and with friends (91.9%). The percentage of heterosexual, cisgender students who agreed or strongly agreed that they felt comfortable expressing their gender identity at home was 93.7%, at school 92.8%, and with friends 94.4%. The percentage of heterosexual, cisgender students who agreed or strongly agreed that they felt comfortable with their sexual orientation at home was 91.2%, at school 89.5%, and with friends 91.9%.

### Self Determination, Bullying, and Mental Health

There were significant main effects for identity and GSA awareness on self-determination, victimization, and symptoms of depression. The main effects of identity revealed that LGBTQ2S+ students scored significantly higher than heterosexual, cisgender students on the victimization subscale of the Illinois Bullying Questionnaire [*F* (1,132) = 10.59, *p* = .001, partial *n*2 = 0.07] and the CESD depression scale [*F* (1,132) = 16.21, *p* < .001, partial *n*2 = 0.11] (see Table 3 for means). With the Self-Determination Basic Psychological Needs Satisfaction Scale, LGBTQ2S+ students scored significantly lower than heterosexual cisgender students on the autonomy, competence, and relatedness subscales [*F* (1,132) = 8.34, *p* = .005, partial *n*2 = 0.06; *F* (1,132) = 4.65, *p* = .033, partial *n*2 = 0.03; *F* (1,132) = 3.97, *p* = .048, partial *n*2 = 0.29; respectively] (see Table 4 for means). For the Basic Psychological Needs Satisfaction in Relationship Scale for teachers, LGBTQ2S+ students scored significantly lower than heterosexual cisgender students on the autonomy and relatedness subscales [*F* (1,132) = 3.97, *p* = .049, partial *n*2 = 0.03; *F* (1,132) = 5.14, *p* = .025, partial *n*2 = 0.04; respectively]. Similarly, LGBTQ2S+ students also scored significantly lower than heterosexual cisgender students on the autonomy and relatedness subscales regarding their feelings of self-determination with family [*F* (1,132) = 11.93, *p* = .001, partial *n*2 = 0.08; *F* (1,132) = 7.00, *p* = .009, partial *n*2 = 0.05; respectively] (see Table 4 for means).

**Table 3.** Means for Bullying and Depression Scales.

	IBQ victimization subscale (*SD*)	CESD (*SD*)
LGBTQ2S+	7.32 (4.40)	29.00 (8.55)
Heterosexual and cisgender	5.46 (2.03)	21.98 (7.09)
*p*-Value	.001	<.001

*Note.* IBQ = Illinois Bullying Questionnaire; CESD = center for epidemiological studies.

**Table 4.** Means for Self Determination Subscales.

	SDS autonomy (*SD*)	SDS competence (*SD*)	SDS relatedness (*SD*)	SDTR autonomy (*SD*)	SDTR relatedness (*SD*)	SDFR autonomy (*SD*)	SDFR relatedness (*SD*)
LGBTQ2S+	4.44 (0.89)	4.07 (1.09)	5.21 (0.98)	4.18 (1.25)	3.38 (1.08)	4.74 (1.49)	5.13 (1.52)
Heterosexual and cisgender	4.81 (0.93)	4.76 (1.05)	5.66 (0.88)	4.81 (1.35)	4.09 (1.26)	5.45 (1.36)	5.80 (1.20)
*p*-Value	.005	.033	.048	.049	.025	.001	.009

*Note. p*-Values are of significant differences between the means for each column. SDS = Self Determination Scale; SDTR = Self Determination Scale Teacher Relationships; SDFR = Self Determination Scale Family Relationships.

The main effect of GSA awareness revealed that students who were aware of their school’s GSA club scored significantly lower on the bullying subscale of the Illinois Bullying questionnaire than the students who were not aware of their school’s GSA [*F* (1,132) = 9.76, *p* = .002, partial *n*2 = 0.07]. Additionally, students who were aware of their school’s GSA club scored significantly higher on the autonomy and relatedness subscales of the Self-Determination Relationships with Family than the students who were not aware of their school’s GSA [*F* (1,132) = 4.92, *p* = .028, partial *n*2 = 0.04; *F* (1,132) = 3.69, *p* = .057, partial *n*2 = 0.03, respectively] (Table 5 for means). The findings did not reveal statistically significant interactions between students’ identity, GSA awareness, bullying experiences, depression, or self-determination.

**Table 5.** Means for Self Determination and Bullying Subscales.

	SDS autonomy (*SD*)	SDFR autonomy (*SD*)	SDFR relatedness (*SD*)	IBQ bullying (*SD*)
Aware of GSA	4.82 (0.93)	5.40 (1.47)	5.76 (1.36)	11.65 (3.09)
Not aware of GSA	4.45 (0.89)	4.96 (1.30)	5.36 (1.16)	13.30 (4.35)
*p*-Value	.008	.028	.03	.002

*Note. p*-Values are of significant differences between the means for each column. SDS = Self Determination Scale; SDFR = Self Determination Scale Family Relationships; IBQ = Illinois Bullying Questionnaire.

## Discussion

The present study investigated the relationship between high school students’ identity, awareness of a GSA, and outcomes of well-being and self-determination. Specifically, we examined students’ experiences of bullying, symptoms of subclinical depression, levels of self-determination, and relationships at home, school, and with their friends. To our knowledge, this study is the first to examine self-determination in relation to school-based GSA clubs.

The first research question examined how comfortable students felt expressing their gender identity and sexual orientation at home, school, and with their friends. A stark finding of our research is how comfortable students felt with their identity in various environments. Specifically, LGBTQ2S+ students felt less comfortable than their heterosexual peers with their sexual orientation at home and school. Interestingly, LGBTQ2S+ students did not express significantly different levels of comfort with their sexual orientation around their friends compared to heterosexual, cisgender students. This supports the notion that friends play an essential role in creating feelings of safety for students. Developing an identity is central to healthy adolescent development. Our data suggest that LGBTQ2S+ students struggle more than their heterosexual, cisgender peers expressing their sexual orientation at school and home. We interpret our results cautiously as the study required parental consent, which has impacted students’ participation in LGBTQ2S+ research, preventing the involvement of adolescents with less family support (Cwinn et al., 2021).

The second research question examined whether students’ identity and awareness of GSAs contributed to experiences of bullying, symptoms of depression, self-determination, and relationships with peers, teachers, and family. LGBTQ2S+ students scored higher than their heterosexual, cisgender peers on symptoms of depression and the victimization subscale of our bullying measure. These findings are consistent with previous research showing that LGBTQ+ students are at higher risk for experiencing bullying and victimization than non-LGBTQ+ students (Cénat et al., 2015; Marx & Kettrey, 2016) and disproportionately suffer from depression and anxiety compared to their non-LGBTQ+ counterparts (Toomey et al., 2011).

The study findings indicated that most students, regardless of their identity, were aware of their school’s GSA, and such awareness was associated with fewer bullying experiences. It may be that knowledge of GSA resources in a school is related to less school-wide bullying. Similarly, Ioverno et al. (2016) found that participants who reported a GSA at their school at the beginning of the academic year also reported fewer experiences of homophobic bullying throughout the year. The findings for bullying are also consistent with other studies that highlight the protective effects of the presence of GSA clubs on all students (Li et al., 2019). The present study focused on the awareness of GSAs. It assessed the experiences of all students (not just LGBTQ+ students) because the presence of a GSA without actively participating in one may benefit all students. Furthermore, involving all students in the study includes those who may not be “out” or navigating their identity without explicitly stating so.

The present study examined self-determination and the basic psychological needs for satisfaction in general and relationships with teachers, peers, and family. An individual’s innate psychological needs of autonomy, competence, and relatedness are the basis for self-motivation and psychological well-being (Deci & Ryan, 2000). The study results highlighted that LGBTQ2S+ students’ psychological needs for autonomy, relatedness, and competence were less fulfilled than their heterosexual, cisgender peers. More specifically, LGBTQ2S+ students reported less autonomy and relatedness with their teachers and members of their families. Interestingly, students aware of their school’s GSA club scored higher on self-determination among multiple subcomponents: general autonomy, autonomy within family relationships, and relatedness with family members. One can speculate that schools with GSAs may be more inclusive, increasing a student’s sense of belonging and that natural process of self-motivation that promotes individual autonomy. Consequently, it is possible that knowledge of GSAs could improve students’ self-understanding and exploration of identities, enhancing their communication, autonomy, and relatedness with family. These results provide insight into the impact and visibility of student-led clubs, such as GSAs in high schools, on the general student population.

### Limitations

This study’s small sample sizes of different sexual orientations and gender identities are a limitation. To compare identity groups, we had to collapse all the sexual orientations and gender identities into one group labeled LGBTQ2S+. Additionally, all the trans and non-binary participants also identified as lesbian, gay, bisexual, pansexual, or an identity other than heterosexual; therefore, we wanted their experiences regarding their sexual orientation to be included in our analysis. We acknowledge that individual students’ experiences vary across different sexual orientations and gender identities. Grouping all LGBTQ2S+ students together may not accurately represent the specific influence of non-cisgender gender identity on school experiences.

The study conclusions are limited to city-specific generalizations that aim to provide a starting place to delve into the nuances of students’ unique experiences based on additional intersecting aspects of race, culture, religion, socio-economic status, and many more. Future research with larger groups of different gender identities and sexual orientations is needed to investigate interactions between gender, identity, and other factors.

The alpha level for the Competence Subscale of The Basic Psychological Needs Satisfaction in Relationship Scale (La Guardia et al., 2000) is a measurement limitation in our study. The reliability was (0.66), which is close to acceptability (.70). It may be that in our research, some items in this subscale did not transfer as well with this sample. Therefore, the findings for this subscale should be interpreted with caution.

The present study included a voluntary sample, and only schools with a functioning GSA participated. Thus, we could not compare findings on questionnaires for schools with GSAs versus schools without GSAs. Future studies could aim to recruit students from schools without a GSA to provide a comparison group and answer future research questions regarding the impact of GSAs on student experiences. Lastly, the participation rate was low (~10%). A caution is that responses from students to our survey do not generalize to an entire population because those who chose not to participate or had parents that did not consent may have had different experiences, backgrounds, or perceived needs than those who did participate. It is possible, for example, that participating students tended to come from more accepting homes and felt more comfortable with their sexual orientation and gender identity.

### Future Directions

The present study reveals an essential aspect of the student experience regarding their identity and self-determination. The findings help highlight that schools still have room for improvement in the socio-contextual conditions that facilitate self-determining behavior and psychological well-being. Future research should aim to capture a rich dialogue from youth that will help us better understand students’ feelings of safety in attending GSA clubs and how participation in GSA clubs can lend to those basic psychological needs of autonomy, relatedness, and competence.

### Relevance to the Practice of School Psychology

This research explores how student-led clubs such as GSAs in high school contexts can impact students’ well-being and inform psychosocial intervention plans that target the school climate. Promoting GSAs in learning environments can create greater school attachment and student connectedness. Increasing the visibility and awareness of these clubs that aim to increase inclusiveness and provide safe spaces for students of gender and sexual minorities can improve mental health and contribute to positive identity development for all students. The results of this study bear directly on the importance of establishing clubs and programs that reduce the risk of coming out at school and enhance LGBTQ2S+ inclusive support. GSAs effectively reduce school challenges for sexual and gender minority students, promote safer environments, and positively influence the school experiences of LGBTQ2S+ and all students.
